# First detection and molecular characterisation of a pigeon aviadenovirus A and pigeon circovirus co‐infection associated with Young Pigeon Disease Syndrome (YPDS) in Turkish pigeons (*Columba livia* domestica)

**DOI:** 10.1002/vms3.662

**Published:** 2021-11-02

**Authors:** Ismail Sahindokuyucu, Merve Biskin Turkmen, Tugce Sumer, Ahmed Eisa Elhag, Mehmet Eray Alcigir, Zafer Yazici, Gerald Barry, Mustafa Yavuz Gulbahar, Oguz Kul

**Affiliations:** ^1^ Poultry Diseases Diagnostic Laboratory, Bornova Veterinary Control Institute Ministry of Agriculture and Forestry Izmir Turkey; ^2^ Faculty of Veterinary Medicine Department of Veterinary Pathology, Kirikkale University Kirikkale Turkey; ^3^ Faculty of Veterinary Medicine Department of Veterinary Virology, Ondokuz Mayis University Samsun Turkey; ^4^ Faculty of Veterinary Sciences Department of Preventive Medicine and Clinical Studies, University of Gadarif Al Qadarif Sudan; ^5^ Veterinary Science Center, School of Veterinary Medicine University College of Dublin Belfield Dublin Ireland; ^6^ Faculty of Veterinary Medicine Department of Veterinary Pathology, Ondokuz Mayis University Samsun Turkey

**Keywords:** co‐infection, isolation, PCR, phylogenetic analysis, pigeon aviadenovirus, pigeon circovirus

## Abstract

Pigeon aviadenovirus A and Pigeon circovirus are both DNA viruses, infect and cause severe clinical diseases in pigeons. These viruses are associated with an immunosuppression syndrome similar to ‘Young Pigeon Disease Syndrome’ (YPDS). This study reports the identification of a natural co‐infection, with severe clinical signs (crop vomiting, watery diarrhoea, anorexia and sudden death) of Pigeon aviadenovirus A and Pigeon circovirus in a breeding pigeon flock in Central Anatolia, Turkey. Both viruses were isolated from pigeons pooled internal organs using primary chicken embryo kidney cell cultures (CEKC) and specific pathogen‐free (SPF) embryonated chicken eggs. Also, both viruses were identified by PCR amplification followed by Sanger sequencing whereas histopathological examination showed degenerated hepatocytes with basophilic intranuclear viral inclusions. As known, both viruses typically have similar transmission characteristics and common clinical manifestations; however, co‐infection may exacerbate the disease with devastating outcomes. This is the first report of its kind in Turkey for those viruses and is essential for the protection against these kinds of infections in pigeons.

## Graphical abstract

Pigeon aviadenovirus A and pigeon circovirus are both DNA viruses, infect and cause severe clinical diseases in pigeons. This study identified a natural co‐infection of both viruses in a breeding pigeon flock in Central Anatolia, Turkey for the first time. Both viruses were isolated from pigeons pooled internal organs using primary chicken embryo kidney cell cultures (CEKC) and specific pathogen‐free (SPF) embryonated chicken eggs. Also, they were identified by PCR amplification followed by Sanger sequencing, whereas histopathological examination showed degenerated hepatocytes with basophilic intranuclear viral inclusions. The viruses typically have similar transmission characteristics and common clinical manifestations, but co‐infection may exacerbate the disease. This is the first report of its kind in Turkey for these viruses and is vital for the protection against such type of diseases in pigeons.

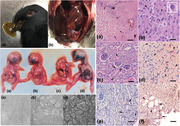

## 1 INTRODUCTION

Viral infections of pigeons, particularly under the age of 1 year old, are associated with high morbidity and mortality. It has been reported that Pigeon aviadenovirus A (PiAdV‐A) (De Herdt et al., 1995; Duchatel et al., 2000) and pigeon circovirus (PiCV) (Todd, 2000) are associated with an immunosuppression syndrome similar to ‘Young Pigeon Disease Syndrome’ (YPDS). A multifactorial infection involves other crucial influencers like stressful conditions and other opportunistic pathogens, especially *Escherichia* (E.) *coli* (Raue et al., 2005). Flock morbidity and mortality for YPDS occur in juvenile pigeons only with up to 20 %; a rate considered lower when compared to other fatal infections in young and adult domestic pigeons like rotavirus A (RVA)‐associated disease, in which flock morbidity is usually high and can reach up 100% (Schmidt et al., 2021).

PiAdV‐A is a member of the genus Aviadenovirus within the family *Adenoviridae*. To date, the Aviadenovirus genus is made up of 15 different species capable of infecting many avian species (Harrach et al., 2011; Marek et al., 2014b; Raue et al., 2002). Pigeon circovirus is a member of the Circovirus genus, family *Circoviridae* and within this genus, 11 different circoviruses could affect birds (Mankertz et al., 2000; Rosario et al., 2017).

Adenovirus infection of a pigeon was first reported in 1976 (McFerran et al., 1976a). Pigeons infected with PiAdV have subsequently been identified in many parts of the world (Vereecken et al., 1998). Although pigeons of any age can be infected, young pigeons under 1 year of age are particularly severely affected by PiAdV‐A, showing acute watery diarrhoea, vomiting and anorexia. PiAdV‐B affects pigeons of all ages and is characterised by sudden death and intensive hepatic necrosis (De Herdt et al. 1995; Duchatel et al. 2000; Vereecken et al., 1998). The annual pigeon mortality rate is very high, with approximately 30% due to PiAdV, but in some cases, it can reach 100% in pigeon lofts with necrotising hepatitis infections (Vereecken et al., 1998).

PiCV was first diagnosed in Canada in 1986, and it is now considered to have a widespread distribution across the world (Woods et al., 1993). Similar to PiAdV‐A, it predominantly causes disease in younger pigeons, mainly between two months and one year of age, with infection causing a broad spectrum of non‐specific clinical signs including lethargy, weight loss, respiratory distress and diarrhoea (Pare et al., 1999; Takase et al., 1990; Tavernier et al., 2000; Todd, 2000; Woods et al., 1994).

Until recently, it was unclear what effect many aviadenoviruses and circoviruses had on pigeons, but now the impact of those viruses such as the fowl aviadenoviruses (FAdV) is well recognised recently due to their role in the multifactorial infection of YPDS as most of the previous studies pointed to the isolation of both viruses from different samples (Hess, 2013; Stenzel et al., 2012). These FAdV strains can cause gizzard erosion (GE), hydropericardium syndrome (HS) and severe liver damage leading to inclusion body hepatitis (IBH) and serotypes 2, 4, 5, 6, 8, 10 and 12 have been isolated from both diseased and healthy pigeons (Goryo et al., 1988; Hess et al., 1998a; Hess et al., 1998b; McFerran et al., 1976a). To increase our understanding of the aforementioned viruses' role in infecting these birds, we report here the isolation and analysis of both PiAdV and PiCV from a co‐infection in pigeons in Central Anatolia, Turkey.

## 2 MATERIALS AND METHODS

### 2.1 History, gross findings and sampling

A pigeon flock (*n* = 45, 4–5 months old domestic pigeons) had a history of increased mortality (20–25%). According to the pigeons’ owner, the first sign was inappetence and vomiting. This was followed by dark green watery diarrhoea that continued for 2–5 days, with many pigeons died during this time. A total of five dead pigeons were collected after a period of chronic weight loss. Two out of the five birds had severe intestinal ascariasis and mucosal oedema. Systematically sampled tissue samples were fixated in 10% neutral formalin for pathology, and liver, kidney, spleen, gut and pancreas were collected under aseptic conditions for virus isolation and identification.

### 2.2 Preparation of supernatants for analysis

Groups of pigeons’ internal organs (liver, kidney, spleen, gut and pancreas) were pooled and mixed with PBS (Sigma‐Aldrich, St. Louis, MO, USA) containing penicillin (2000 units/ml), streptomycin (2 mg/ml), gentamicin (50 μg/ml) and Mycostatin (1000 units/ml) (Sigma‐Aldrich). The organs were homogenised, followed by centrifugation (4000 rpm for 10 min). The supernatants were then collected for screening by PCR and virus isolation.

### 2.3 Isolation of viruses

#### 2.3.1 Isolation in SPF embryonated chicken eggs

Supernatants of pigeons pooled organs that were positive by PCR for PiAdV‐A and PiCV were first filtered (0.22 μm microfilter) and then inoculated into the chorioallantoic cavity of 10‐day‐old specific pathogen‐free (SPF) embryonated chicken eggs and the yolk sac of 6‐day‐old SPF eggs to observe and compare the viruses propagation according to the protocol of the Villegas Laboratory Manual (Villegas, 2006). The eggs were incubated at 37°C for 5 or 10 days according to the inoculation route, respectively. The inoculated eggs were examined on a daily basis until embryos stopped moving and were presumed dead under ovoscope light. At this point, the allantoic fluid was collected and inoculated into fresh SPF eggs following the same procedure as before. This was repeated until each supernatant was passaged through five eggs.

#### 2.3.2 Isolation in cell culture

Supernatants to be used for inoculation of cell cultures were prepared from pooled organs and screened by PCR as described in the previous section. Primary chicken embryo fibroblast (CEF) and chicken embryo kidney cell (CEKC) cultures were prepared from 10‐day‐old and 18‐day‐old SPF embryonated chicken eggs according to the protocol of the Villegas Laboratory Manual, respectively (Villegas, 2006). For cell propagation, MEM (Gibco, Paisley, UK) containing Earle's balanced salts, 10% fetal calf serum (Gibco), 100 IU of penicillin (Sigma‐Aldrich) and 100 μg of streptomycin (Sigma‐Aldrich) per ml was used. The cells were incubated at 37°C for 24–48 h until a confluent monolayer of CEFs or CEKCs had formed. Cell culture supernatants were then removed and filtered supernatants were overlaid onto the cell cultures, then incubated at 37°C for 1 h. Following this, fresh MEM supplemented with 2% fetal bovine serum (Gibco) was added to the cultures, and they were incubated at 37°C in 5% CO_2_ for 7 days. Once evident, widespread CPE was observable, the cultures were freeze‐thawed, centrifuged at 4000 × *g* for 10 min, and the supernatants were stored at −20°C until DNA extraction was performed.

### 2.4 Pathologic examination

#### 2.4.1 Necropsy and histopathological examinations

After necropsy, multiple tissue samples were fixed in 10% neutral formalin (pH 7.4) and processed according to routine tissue processing procedures. After embedding in paraffin wax, tissue sections were cut at 5‐μm thickness from paraffin blocks and stained with haematoxylin and eosin (H&E) (Luna, 1968). The histopathological changes in the lesioned organs were semiquantitatively scored by a trained veterinary pathologist using a brightfield microscope and their photomicrographs were taken (Olympus BX51, DP25 digital camera). The semiquantitative scoring system used was as follows: (−) none, (+) mild, (++) moderate and (+++) severe.

#### 2.4.2 Fowl aviadenovirus‐4 immunoperaxidase test

The avidin‐biotin complex peroxidase (ABC‐P) method was carried out according to the manufacturer's instructions (ab64264, Abcam, HRP/DAB, Waltham, MA, USA). Briefly, tissue sections were deparaffinised, hydrated and then boiled in antigen retrieval solution (citrate buffer pH 6.0) for 20 min. The sections were incubated in a 3% hydroperoxide‐methanol mixture at room temperature for 20 min before blocking was performed using normal goat serum at 37°C for 7 min. The sections were incubated with anti‐FAdV‐4 hyperimmune serum produced in rabbits kindly provided by Dr. Hafez Mohamed Hafez (Freie Universität Berlin‐Germany) and diluted at 1:40 at 37°C for 1 h. After this, the sections were incubated with a biotinylated, HRP labeled anti‐rabbit secondary antibody (Abcam kit, Waltham, MA, USA) followed by exposure to the AEC substrate chromogen solution (Dako, Santa Clara, CA, USA). For counterstaining, sections were kept in Gill's haematoxylin (Sigma‐Aldrich) for 2 min and coverslipped with an aqueous mounting medium. Washing procedures were carried out with Tris‐HCl; twice for 5 min, excluding the blocking step. The sections were treated with phosphate buffered saline (PBS) instead of primary antibodies as a negative control method for the test. As a positive control, the same protocol was performed on chicken liver and brain sections from known FAdV‐4‐infected chickens, which were kindly supplied by Dr. Hafez Mohamed Hafez (Freie Universität Berlin‐Germany). The ABC‐P test results were semiquantitatively scored by a trained veterinary pathologist using a brightfield microscope and their photomicrographs were taken (Olympus BX51, DP25 digital camera). The semiquantitative scoring system used was as follows: (−) none, (+) mild, (++) moderate and (+++) severe.

### 2.5 DNA extraction and PCR

DNA was extracted from 200 μl aliquots of prepared supernatants (prepared as described previously) or from cell cultures using a High Pure Viral Nucleic Acid Kit (Roche, Basel, Switzerland), according to the manufacturer's protocol. The extracted DNAs were stored at −20°C until PCR was performed.

The PCRs for PiAdV‐A and PiCV were carried out in total volumes of 25 μl containing 5 μl of DNA, 0.4 μM of each primer and PCR master mix (Grisp, Porto, Portugal). The PCR for PiAdV‐A was carried out in a thermocycler (Techne, Essex, UK) with an initial denaturation at 95°C for 5 min, followed by 35 cycles of 95°C for 20 s, 58°C for 30 s and 72°C for 1 min, with a final elongation step at 72°C for 5 min. The PCR for PiCV was carried out in the same thermocycler as the PiADV‐A PCR with an initial denaturation at 95°C for 5 min, followed by 35 cycles of 95°C for 20 s, 62°C for 30 s and 72°C for 30 s, with a final elongation step at 72°C for 5 min. Before Sanger sequencing, the PCR amplicons were visualised by agarose gel electrophoresis and then purified according to the manufacturer's protocol (ExoSAP‐IT PCR Product Cleanup Reagent, Thermo Fisher Scientific, Waltham, MA, USA). All primers used in this study are listed in Table 1.

**TABLE 1:** Information about the primers used in this study

Viruses	Target gene	Primer names	Sequence (5′–3′)	Product size (bp)	Reference numbers
FAdV	Hexon	Hexon A	CAARTTCAGRCAGACGGT	897	(Meulemans et al., 2001)
		Hexon B	TAGTGATGMCGSGACATCAT		
FAdV	Hexon	H1	TGGACATGGGGGCGACCTA	1219	(Raue & Hess, 1998)
		H2	AAGGGATTGACGTTGTCCA		
FAdV	Hexon	H3	AACGTCAACCCCTTCAACCACC	1319
		H4	TTGCCTGTGGCGAAAGGCG		
PiAdV‐A	Fibre‐2	PiAdV‐A F1‐s	ATCAACTACGACAACGAAGGC	967	(Raue et al., 2002)
		PiAdV‐A F2‐as	CGGTAGAGTTACGGGGAAATT		
PiAdV‐B	Hexon	PiAdV‐B Hex‐3‐F	GTAACATGAGCGTGCTGTTTG	643	(Teske et al., 2017)
		PiAdV‐B Hex‐3‐R	CTGAGAAACGAAACCCGAATTG		
PiHV	Polymerase	PiHV‐s	GGGACGCTCTGATTAAGGAAT	242	(Raue et al., 2005)
		PiHV‐as	CTTGGTGATCAGCAGCAGCTTG		
PiCV	Capsid	PiCV2‐s	TTGAAAGGTTTTCAGCCTGGC	325	(Freick et al., 2008)
		PiCV2‐as	AGGAGACGAAGGACACGCCTC		

### 2.6 DNA sequencing and phylogenetic analysis

The amplified fibre‐2 gene (PiAdV‐A) and capsid gene (PiCV) amplicons were Sanger sequenced by Microsynth (Balgach, Switzerland) in both forward and reverse directions, using the same PCR primers that used to amplify the products. Sequences were then aligned and edited using MEGA X (Molecular Evolutionary Genetics Analysis, version 10.1.8) software (The Biodesign Institute, Tempe, AZ, USA). The nucleotide sequences of the field isolates presented here (TR/SKPA20 for PiAdV‐A and TR/SKPC20 for PiCV) have been submitted to the National Center for Biotechnology Information (NCBI)‐GenBank database under the accession numbers: MN985817 and MT130538, respectively. The sequence data were then compared to complete genome reference sequences of aviadenovirus and circovirus species available from the (NCBI) and their phylogenetic relationships were investigated using a BLAST search (https://blast.ncbi.nlm.nih.gov). Phylogenetic trees were built using the neighbour‐joining method with the Hasegawa–Kishino–Yano and Kimura‐2 parameter substitution model and gamma distribution using the maximum‐likelihood statistical method in MEGA X, respectively (Hasegawa et al., 1985; Kimura, 1980; Kumar et al., 2018).

## 3 RESULTS

### 3.1 Necropsy and histopathological findings

**FIGURE 1:**
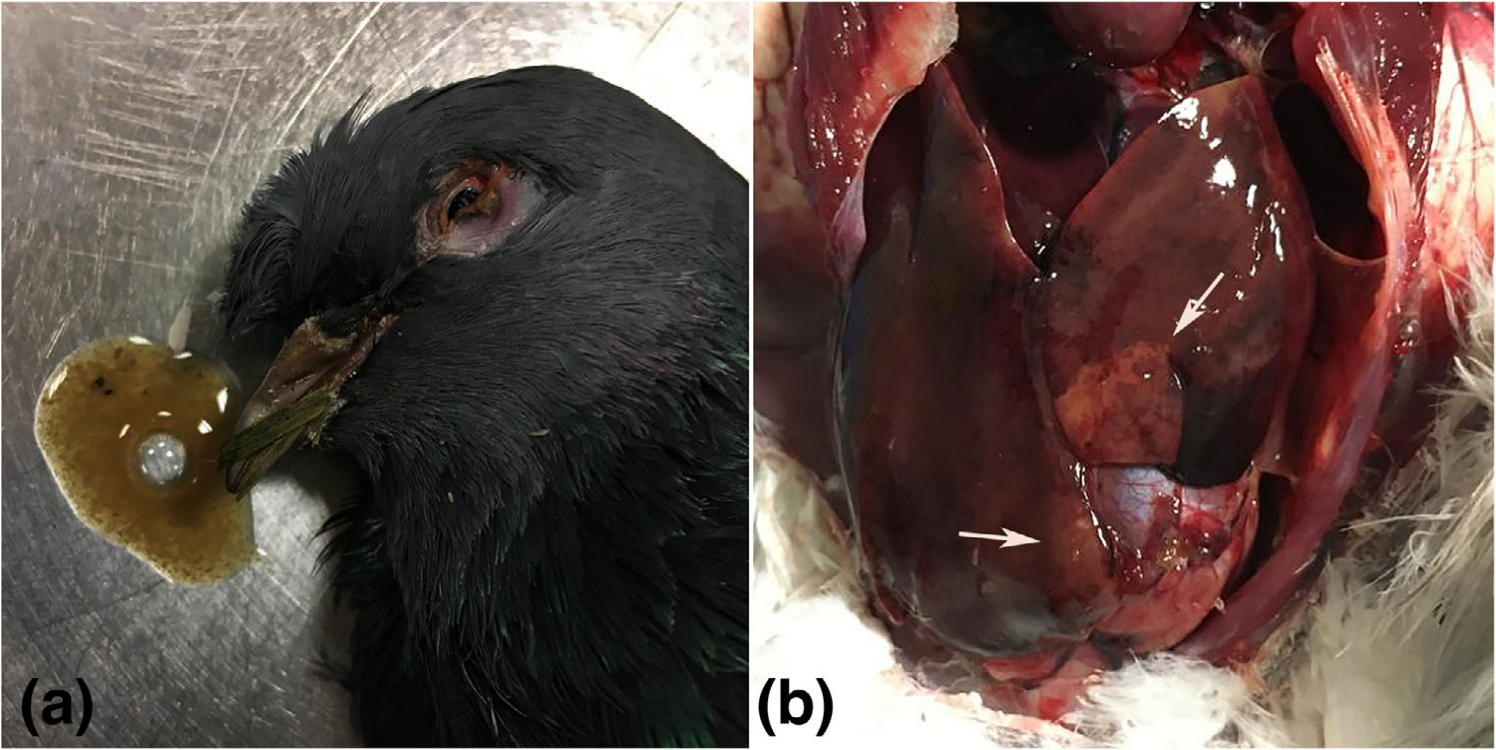
(a) Picture of one of the infected birds that were necropsied. Vomit from the bird is shown beside the bird's head, characterised by mucous and watery content. The bird had shrunken eyes indicating dehydration and poor condition. (b) Image of an example infected pigeon liver taken during necropsy. Widespread discoloration indicates severe necroses (white arrows) and haemorrhages on the liver

Macroscopically, the pigeons were dehydrated and emaciated; pectoral muscle atrophy was prominent and postmortal watery vomit was detected in two pigeons (Figure 1). The most evident gross findings were observed on livers (*n* = 3). Affected livers showed enlargement and fragility associated with pale, grey‐yellow necrotic areas (Figure 1) and widespread haemorrhagic spots. The proventriculus and stomach mucosae were thickened and oedematous in appearance, and in some instances, they were coated with a sticky mucous. The kidneys were enlarged and had multifocal grey foci on the serosal surfaces.

**FIGURE 2:**
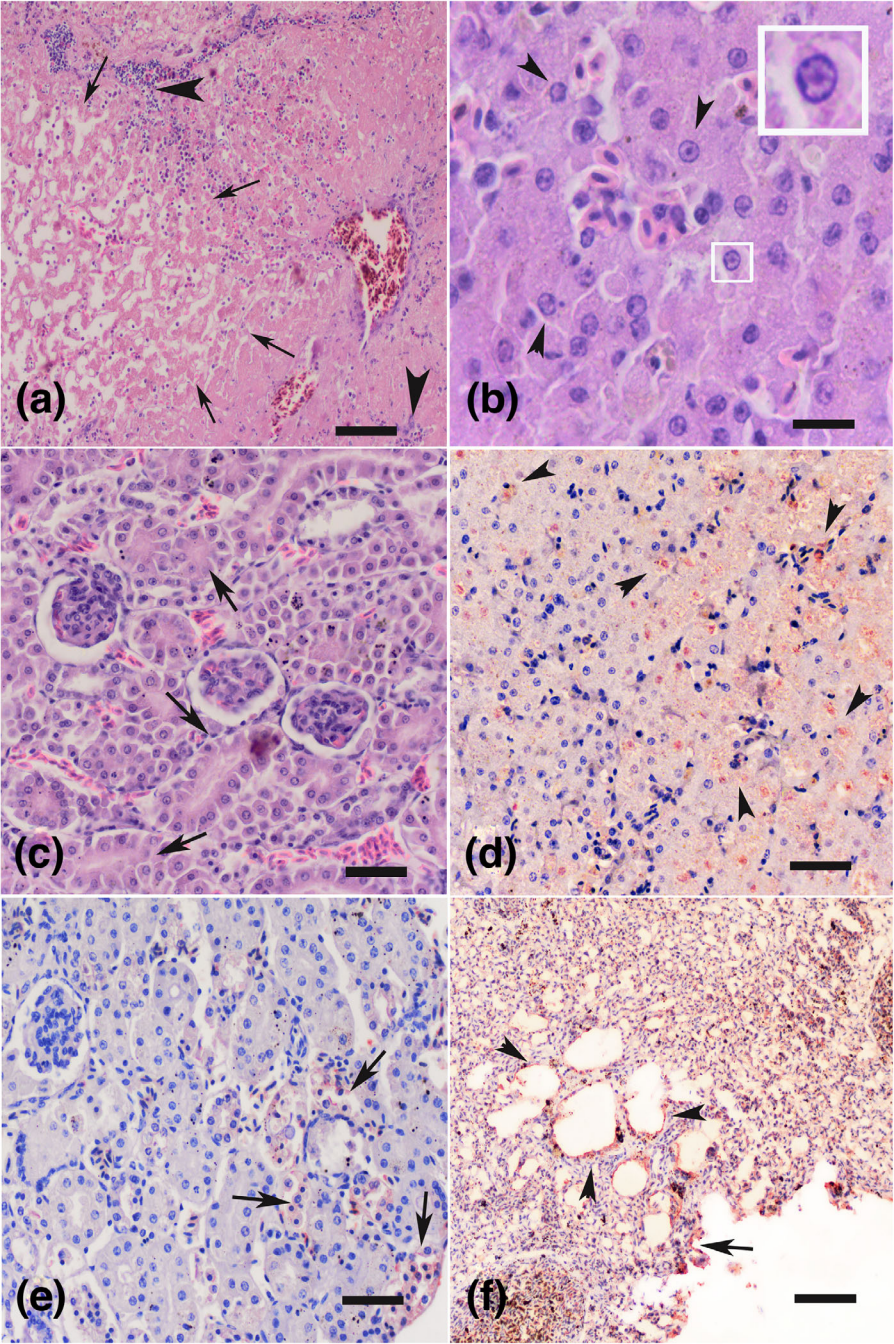
(a) Section of the liver from a necropsied pigeon. Severe coagulation necrosis, homogeneous eosinophilic appearance and some structural remnants of hepatic cords are visible, and the hepatocytes are completely necrotic (arrows). At the periphery, focal lymphocyte and macrophage accumulations (arrowheads) are visible. The section was stained with Haematoxylin and Eosin, bar = 300 μm. (b) Section of the liver from a necropsied pigeon. Clear nuclei are visible due to the characteristic circular margination of the chromatin in the hepatocytes. Some degenerated hepatocytes had basophilic intranuclear viral inclusions (arrowheads and inset). The section was stained with Haematoxylin and Eosin, bar = 45 μm. (c) Section was taken from the kidney of a necropsied pigeon. Hydropic degeneration was visible along with necrosis of the cortical tubule epithelia. In healthy tubular epithelia, the nuclei seemed clear but contained numerous intranuclear basophilic inclusions. The section was stained with Haematoxylin and Eosin, bar = 90 μm. (d) Section was taken from the liver of a necropsied pigeon. Red coloration indicates the presence of FAdV‐4 antigen, detected by the ABC‐P assay. In this figure, red dots are seen in necrotic areas and degenerated hepatocytes (arrowheads). The section was counterstained with Gill's haematoxylin, bar = 180 μm. (e) Section was taken from the kidney of a necropsied pigeon. Red coloration indicates the presence of FAdV‐4 antigen, detected by the ABC‐P assay. In this figure, red dots are seen in the degenerative and necrotic tubule epithelia of the kidney. The section was counterstained with Gill's haematoxylin, bar = 180 μm. (f) Section was taken from the respiratory system of a necropsied pigeon. Red coloration indicates the presence of FAdV‐4 antigen, detected by the ABC‐P assay. This figure shows red coloration on the bronchiolar epithelia (arrowheads) and alveolar walls (arrow). The section was counterstained with Gill's haematoxylin, bar = 300 μm

Histopathologically, there were multifocal coagulation necroses, characterised by acellular homogenous eosinophilic appearance and mononuclear cell infiltrations, surrounded by necrotic cell debris. In severe cases, widespread haemorrhages showed association with hepatic necroses. Hepatocytes predominantly showed parenchymatous degeneration, cytoplasmic swelling and clear hyperchromatic nuclei, while intranuclear basophilic viral inclusions were also observed in some cells (Figure 2). In the kidneys, multifocally localised interstitial lymphocytes, plasma cell infiltrations in the cortex and tubular hydropic degenerations were all observed. In two cases, interstitial pneumonia, alveolar epithelial hyperplasia and bronchiolitis were seen. In the proventriculus mucosae, numerous heterophilic leucocyte infiltrations were present in the submucosae. Immunohistochemistry identified the presence of FAdV‐4 antigen in degenerated hepatocytes, kidney tubule epithelia, interstitial inflammatory cells and urothelial epithelia, as well as alveolar and bronchial epithelia in the lungs as seen in Table 2.

**TABLE 2:** Results of the semiquantitative scoring of histopathological and ABC‐P test findings. IPT: Immunoperoxidase test

	Severity of histopathological findings			
Case#	Liver	Kidneys	Lungs	IPT (FAdV‐4)
1	+++	++	−	+++
2	++	+++	−	++
3	+	+	−	+
4	++	+	+	++
5	+++	++	−	+++

### 3.2 Virus isolation

**FIGURE 3:**
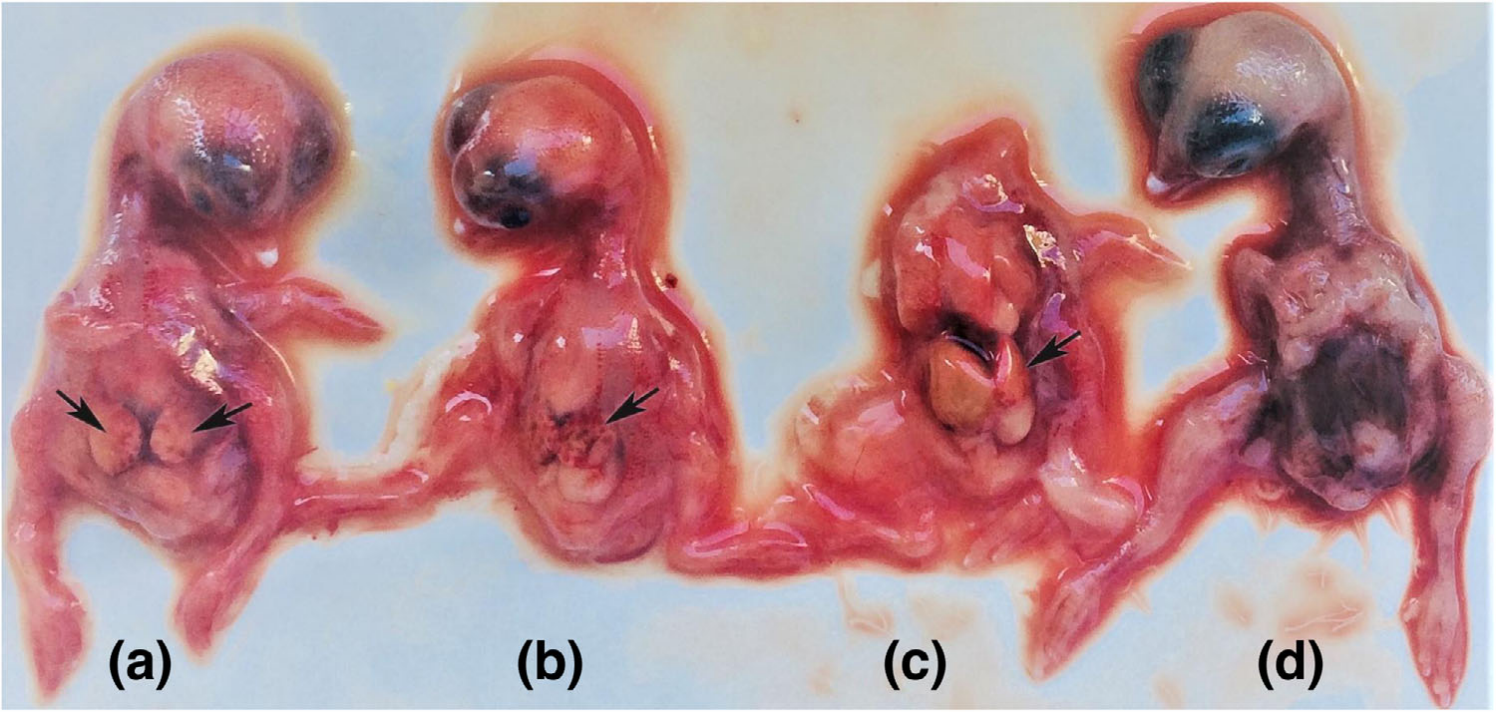
SPF chicken eggs were inoculated with PCR positive supernatants for PiCV (TR/SKPC20). The eggs were incubated for either 5 or 10 days depending on the inoculation route (See methods section) before removal of the embryos and examination. This figure show examples of the embryos seen. Livers with multiple petechial haemorrhages (black arrows), extensive malformations and haemorrhaging were all visible in embryos from the PiCV (PCR positive) inoculated eggs (a, b, c) and these features were not present in embryos from mock‐inoculated eggs (d)

The embryos were monitored every day by visualisation using an ovoscope light. Embryos started to die approximately 3 days after inoculation (for both routes, see Figure 3). After five passages in eggs, the degenerated and dead embryos’ internal organs, as well as the chorioallantoic fluid, were then collected and tested by PCR to confirm the presence of different viruses; also, the chorioallantoic fluid was tested by haemagglutination assay to assess the presence of avian viruses such as Newcastle disease or avian influenza viruses. All PCR assays were negative except for the PCR test that used primers specific for PiCV. The haemagglutination assay was negative as well for any virus that could cause haemagglutination.

**FIGURE 4:**
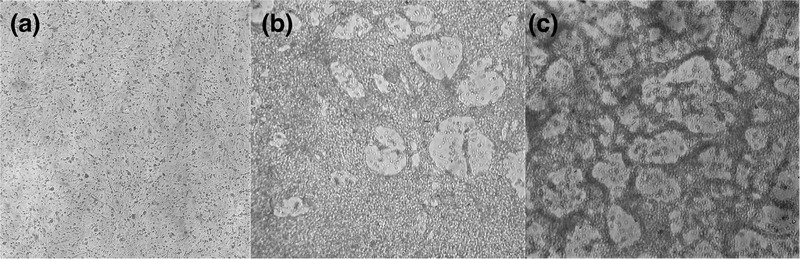
Both the PiAdV‐A (TR/SKPA20) and PiCV (TR/SKPC20) isolates caused cytopathic effects (CPE) in CEKC cultures. (a) Mock‐infected CEKCs with no CPE visible. (b) CEKC culture inoculated with a PiAdV‐A isolate and (c) CEKC culture inoculated with a PiCV isolate. Cultures b and c show rounding and detachment of cells leading to apparent damage to the monolayer. Images were taken 72 h post‐inoculation

After inoculating the supernatants into two different cell cultures, there was no visible cytopathic effect in primary CEFCs. In contrast, supernatants caused extensive rounding, clumping and detachment of cells 48–72 h post‐inoculation in the CEKC cultures (Figure 4). DNA extracted from these cultures was tested by PCR and was positive for both PiCV and PiAdV‐A, suggesting the growth of both viruses in the culture together. At this point, the PiAdV‐A isolate was named ‘TR/SKPA20’, and the PiCV isolate was named ‘TR/SKPC20’.

### 3.3 PCR and phylogenetic analysis of the isolates

**FIGURE 5:**
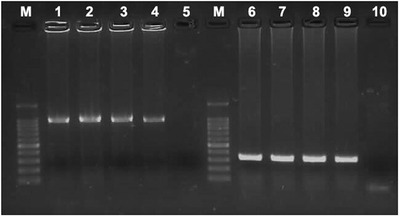
Agarose gel showing the results of PCR with different pigeon tissue supernatants, using primers specific for PiAdV‐A (lane 1 to 4; band is at approximately 967 bp) and PiCV (lane 6 to 9; band is at approximately 325 bp.) Lane M, 100 bp ladder marker; lane 1, TR/SKPA20 (liver); lane2, TR/SKPA20 (kidney); lane 3, TR/SKPA20 (spleen); lane 4, TR/SKPA20 (gut and pancreas); lane 5, negative control. Lane 6, TR/SKPC20 (liver); lane7, TR/SKPC20 (kidney); lane 8, TR/SKPC20 (spleen); lane 9, TR/SKPC20 (gut and pancreas); lane 10, negative control

After initially screening the supernatants generated from pigeon tissues by PCR for several different viruses, the only positive hits were for parts of the PiAdV‐A fibre‐2 and PiCV capsid genes (Figure 5).

**FIGURE 6:**
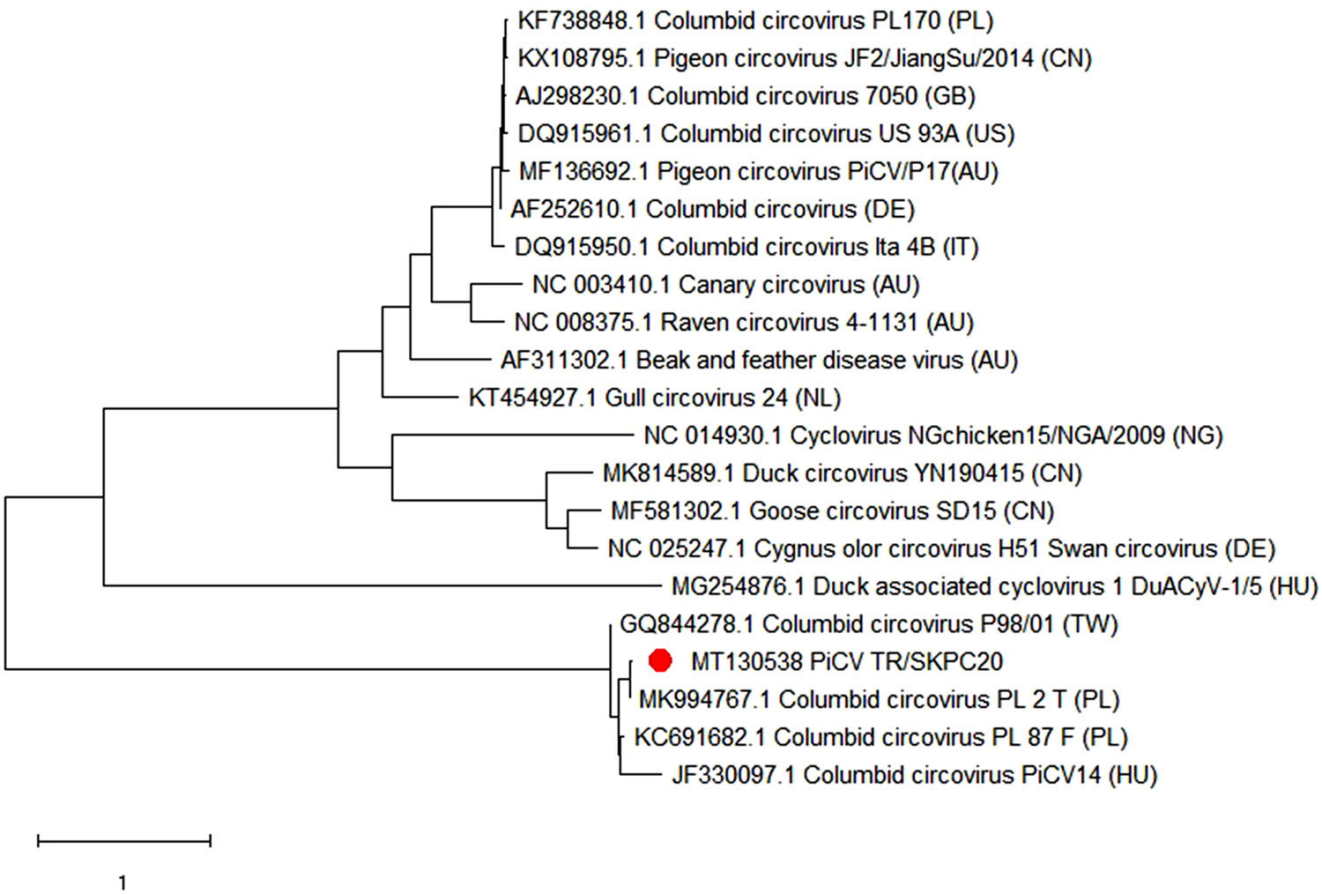
Phylogenetic tree based on the amino acid sequence of the PiCV capsid gene. The tree shows circovirus species that affect fowl. The tree shows the names and GenBank accession numbers of each isolate. A red dot indicates the PiCV isolate from this study

**FIGURE 7:**
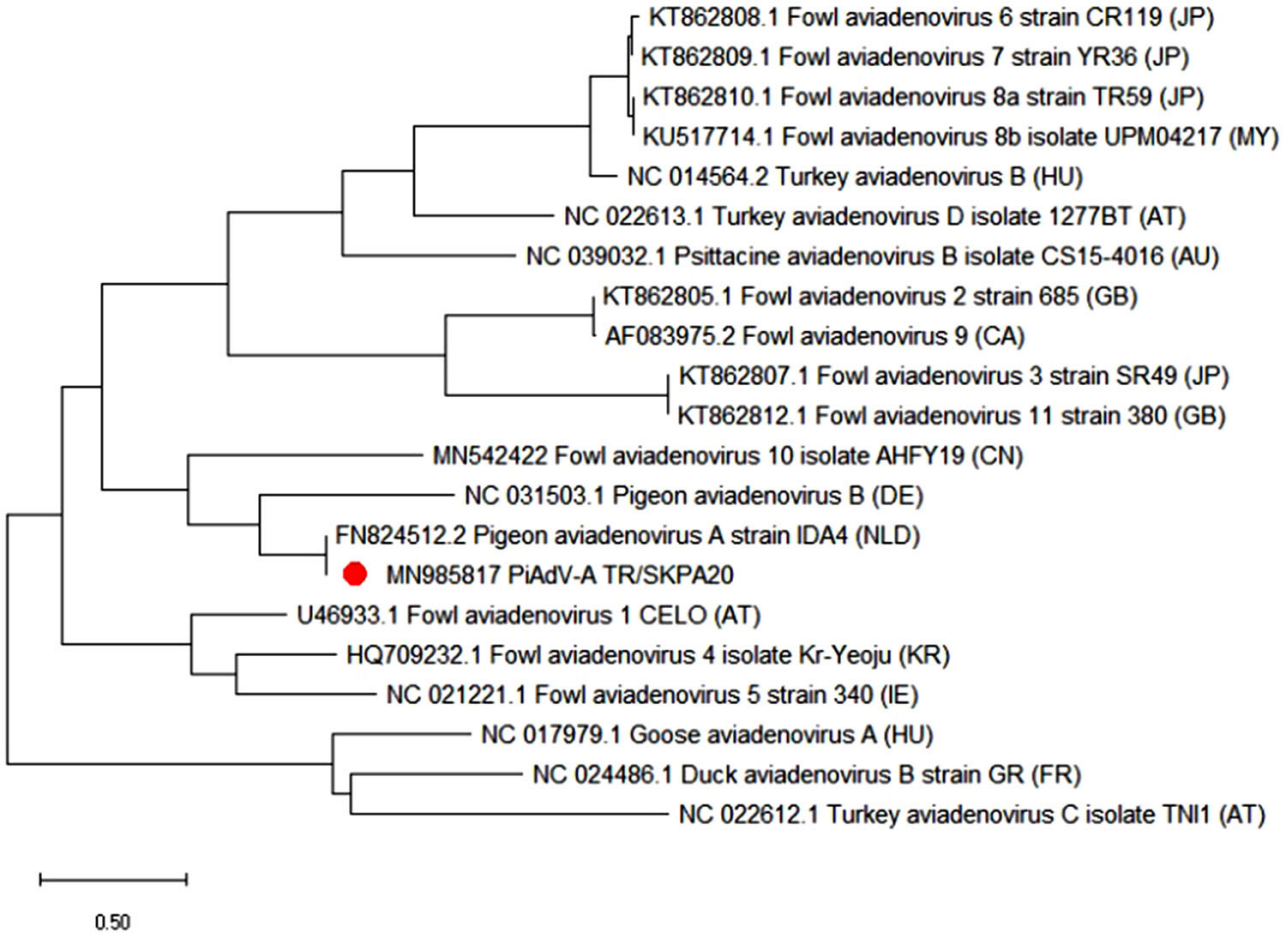
Phylogenetic tree based on the amino acid sequence of the PiAdV fibre‐2 gene. The tree shows aviadenovirus species that affect fowl. The tree shows the names and GenBank accession numbers of each isolate. The PiAdV‐A isolate from this study is indicated by a red dot

As a result of the BLAST search, the PiCV field isolate, TR/SKPC20, was found to be most closely related to strains previously isolated in Poland (MK994767, KC691682), Hungary (JF330097) and Taiwan (GO844278) (Figure 6). The PiAdV‐A field isolate, TR/SKPA20, was found to be most closely related to a previously sequenced PiAdV‐A strain called IDA4, with 99.03% similarity at the amino acid level (Figure 7).

## 4 DISCUSSION

In the present study, we are reporting a PiCV, PiAdV co‐infection associated with severe clinical signs (crop vomiting, watery diarrhoea, anorexia and sudden death) in a flock of pigeons in Kirikkale province, Turkey; both viruses, individually, have been previously associated with known diseases that affect pigeons all over the world (De Herdt et al., 1995; Schmidt et al., 2008).

Pathological examination of the pigeons revealed severe, systemic pathological changes with a particular focus on the liver. An immunoperoxidase assay was performed on tissue sections using a serum containing antibodies that detect a common group‐specific antigen of the 12 fowl adenovirus serotypes (Hess, 2000; Wan et al., 2018). The assay highlighted the presence of fowl adenovirus antigen in lesions that were visible in tissue sections from different birds that had undergone necropsy. However, because of the cross‐reactivity of these antibodies, it was necessary to use molecular techniques and virus isolation to confirm precisely what virus was present (Hess, 2000).

PCR was used to screen tissue samples from the pigeons for multiple viruses and primers based on the PiAdV‐A fibre‐2 gene (Raue et al., 2002) and PiCV capsid gene (Freick et al., 2008), and the amplified fragments were sequenced and identified as PiCV and PiAdV‐A. Unusual to previous reports, our obtained PCR test results showed that the internal organs like liver, kidney, spleen, gut and pancreas had an equal affinity of PiCV and PiAdV‐A. To the best of our knowledge, this study reports the first identification and isolation of PiAdV‐A and PiCV in a pigeon flock in Turkey, which may point to the significance of both viruses in evoking immunosuppression and severity of YPDS; also, these results could help in understanding those viruses role in the pathogenesis of the YPDS in consist with our clinical findings, thus assist veterinary authorities in implementing effective control plans to prevent local pigeons flocks from this devastating syndrome.

Following PCR identification, both PiAdV‐A and PiCV were successfully cultured in CEKC cultures, while PiCV could also be cultured in SPF chicken eggs. Inoculating eggs with PiCV supernatants led to the death of the embryos present, suggesting infection and spread of the virus through the embryos. Primary CEF cell cultures were also used to isolate the suspected viruses; however, these propagation attempts were unsuccessful, suggesting that the viruses supposed to be present were either not infecting or not replicating in those cells. Although PiCV is regularly grown in culture, PiAdV serotypes are reported to be quite challenging to grow in cell culture (Vereecken et al., 1998; Duchatel et al., 2000; Marlier & Vindevogel, 2006; Schmidt et al., 2008). The isolation of different pigeon avidenovirus strains has previously been achieved in primary CEKC and primary CELC (McFerran et al., 1976a), but the conditions used in this study were not suitable or optimal and further work is required (Raue & Hess, 1998; Takase et al., 1990; Vereecken et al., 1998). Despite this, both isolates are now available for further molecular and virological studies. Relatively little work has been carried out on either of these viruses and there is now great potential for analysis to be pursued. For example, little is understood about the fibre‐2 protein of PiAdV‐A, but the fibre protein of FAdV is known to play a crucial role in viral infection and pathogenesis (Lu et al., 2019; Pallister et al., 1996; Zhang et al., 2018). It is responsible for the initial attachment of the virus to cellular receptors, including the involvement of cell surface integrins (Wickham et al., 1993) and it is possible that the fibre‐2 protein carries out a similar function. Considering the importance of these proteins and their involvement in infection, they may also play a role in species specificity and cross‐species infection.

It is known, for example, that several FAdV serotypes 2, 4, 5, 6, 8, 10 and 12 have been previously isolated from diseased and healthy pigeons (Goryo et al., 1988; Hess et al., 1998a; Hess et al., 1998b; McFerran et al., 1976a). In this present study, other viruses such as FAdV (1 to 7, −8a and −8b, −9 to 11) and PiAdV‐B and PiHV were all screened for but not detected (Table 1). In a recent study, concerning adenoviral poultry diseases, including body hepatitis (IBH), were reported for the first time in broiler and broiler breeder flocks in Turkey (Sahindokuyucu et al., 2020). PiAdV‐A in pigeons is sometimes called IBH due to the intranuclear basophilic bodies seen under histopathology examination (Abadie et al., 2001; McFerran et al., 1976b), and it is possible that misdiagnosis has previously occurred and cross‐species infections are already occurring. Considering the ability of these viruses to be highly pathogenic in pigeons, it will be crucial going forward to understand the similarities between these viruses and other fowl aviadenoviruses that can infect commercially important birds, thus allowing consideration of the potential for these viruses to jump species and cause outbreaks in birds such as chickens in the future.

In a similar study, a total of 107 pigeon flocks were examined and pigeon circovirus (PiCV) genetic material was the most frequently detected, pigeon herpesvirus (PiHV) genetic material was second in frequency, while genetic material of pigeon aviadenovirus was found only in two flocks of young birds with clinical symptoms of YPDS. Moreover, the presence of fowl aviadenovirus (FAdV) genetic material was not detected in any of the studied flocks (Stenzel et al., 2012). Our study reports the first identification and isolation of PiAdV‐A and PiCV in a pigeon flock in Turkey to the best of our knowledge. In contrary to previous studies, PiHV and FAdV genetic material were not detected in the tested flocks.

## 5 CONCLUSION

The clinical signs observed in pigeons of this study would typically be associated with Young Pigeon Disease Syndrome (YPDS), a multifactorial disease in which PiCV has been shown to play an important role. PiCV is associated with immunosuppression, making the birds more susceptible to secondary bacterial, viral or parasitic infections. This study indicates that co‐infections of PiAdV and PiCV are possible and are associated with a severe, sometimes fatal disease in the birds infected. Potentially, an initial PiCV infection weakened the flock, making it more susceptible to a PiAdV infection, and this enhanced the clinical disease that occurred in the birds. While merely speculation, it suggests a possible mechanism that would explain this devastating multi‐pathogen infection in the flock.

It is crucial in the future to measure the prevalence of both viruses in pigeon populations to determine their pathogenic burden. Experimental co‐infections to tease apart the pathogenesis of disease will be essential so that if this happens more frequently or starts to create problems in commercially important fowl, then it can be recognised quickly and tackled effectively. This study paves the way for these future studies and offers the potential for a better understanding of viral infections of birds in Turkey.

## ETHICS STATEMENT

The authors confirm that the ethical policies of the journal, as noted on the journal's author guidelines page, have been adhered to. No ethical approval was required, as all sampling was carried out on birds that had died as a result of the virus infection. No birds were culled or treated during this study.

## FUNDING

No funding or financial support either locally or internationally was received for this study.

## AUTHOR CONTRIBUTIONS

**Ismail Sahindokuyucu**: Conceptualisation; methodology; writing – original draft preparation. **Merve Biskin Turkmen**: Investigation; methodology. **Tugce Sumer**: Investigation; methodology. **Ahmed Eisa Elhag**: Formal analysis; visualisation; writing – original draft preparation. **Mehmet Eray Alcigir**: Investigation; methodology. **Zafer Yazici**: Conceptualisation; supervision; visualisation; writing – review & editing. **Gerald Barry**: Formal analysis; visualisation; writing–review & editing. **Mustafa Yavuz Gulbahar**: Formal analysis; visualisation. **Oguz Kul**: Conceptualisation; resources; supervision; writing – review & editing.

## CONFLICT OF INTEREST

The authors declare that there is no potential conflict of interest.

### PEER REVIEW

The peer review history for this article is available at https://publons.com/publon/10.1002/vms3.662

## Data Availability

The data that support the findings of this study are openly available in the GenBank database at https: //www.ncbi.nlm.nih.gov/nucleotide/ under the names: TR/SKPA20 for PiAdV‐A and TR/SKPC20 for PiCV and accession numbers: MN985817 and MT130538, respectively.
